# Serotonin receptor gene (*HTR2A*) T102C polymorphism modulates individuals’ perspective taking ability and autistic-like traits

**DOI:** 10.3389/fnhum.2015.00575

**Published:** 2015-10-23

**Authors:** Pingyuan Gong, Jinting Liu, Philip R. Blue, She Li, Xiaolin Zhou

**Affiliations:** ^1^Key Laboratory of Resource Biology and Biotechnology in Western China, Ministry of Education, Northwest UniversityXi’an, China; ^2^Center for Brain and Cognitive Sciences and Department of Psychology, Peking UniversityBeijing, China; ^3^Laboratory of Medical Molecular Biology, Henan University of Science and TechnologyLuoyang, China; ^4^China Center for Special Economic Zone Research, Shenzhen UniversityShenzhen, China; ^5^Research Centre for Brain Function and Psychological Science, Shenzhen UniversityShenzhen, China; ^6^Key Laboratory of Machine Perception (Ministry of Education), Peking UniversityBeijing, China; ^7^IDG/McGovern Institute for Brain Research, Peking UniversityBeijing, China

**Keywords:** empathy, perspective taking, autistic-like traits, serotonin, *HTR2A*

## Abstract

Previous studies have indicated that empathic traits, such as perspective taking, are associated with the levels of serotonin in the brain and with autism spectrum conditions. Inspired by the finding that the serotonin receptor 2A gene (*HTR2A*) modulates the availability of serotonin, this study investigated to what extent *HTR2A* modulates individuals’ perspective taking ability and autistic-like traits. To examine the associations of the functional *HTR2A* polymorphism T102C (rs6313) with individuals’ perspective taking abilities and autistic-like traits, we differentiated individuals according to this polymorphism and measured empathic and autistic-like traits with Interpersonal Reactivity Index (IRI) and Autism-Spectrum Quotient (AQ) scale in 523 Chinese people. The results indicated that this polymorphism was significantly associated with the scores on Perspective Taking and Personal Distress subscales of IRI, and Communication subscale of AQ. Individuals with a greater number of the C alleles were less likely to spontaneously adopt the point of view of others, more likely to be anxious when observing the pain endured by others, and more likely to have communication problems. Moreover, the genotype effect on communication problems was mediated by individuals’ perspective taking ability. These findings provide evidence that the *HTR2A* T102C polymorphism is a predictor of individual differences in empathic and autistic-like traits and highlight the role of the gene in the connection between perspective taking and autistic-like traits.

## Introduction

Empathy, in the broadest sense, is the lens through which we understand, experience, and respond to the internal states of others (Davis, 1983). It is composed of a variety of skills or components, such as perspective taking (the understanding of another person’s beliefs and thoughts, also termed “theory of mind”) and empathic response (the emotional response to others’ affective states) (Baron-Cohen and Wheelwright, 2004; Batson, 2009; Shamay-Tsoory, 2009). These skills allow us to understand and predict others’ motives, intentions, thoughts, and emotions, so as to foster and maintain social relationships (Davis and Oathout, 1987). Deficit in empathic ability is a central feature of social behavioral abnormalities. For example, individuals with autism, which is a developmental disorder characterized by restricted interests, stereotyped and repetitive behaviors, as well as deficits in social interaction and communication (Gmitrowicz and Kucharska, 1994), show severe impairment in perspective taking (Baron-Cohen et al., 1985).

Empathic traits are strikingly variable among individuals. Twin studies showed that empathic traits are highly heritable, with a heritability of up to 67% for perspective taking and 34–47% for empathic response (Hughes and Cutting, 1999; Knafo et al., 2008), implying that individual differences in empathic components are strongly influenced by individuals’ genetic expression. Although previous studies have demonstrated the contribution of genes such as the oxytocin receptor gene and the dopamine beta-hydroxylase gene to individual differences in empathy (Rodrigues et al., 2009; Wu et al., 2012; Gong et al., 2014), most of the genes involved in empathic traits are still under-investigated. In this study, we aimed to examine to what extent the polymorphism of serotonin receptor 2A gene (*HTR2A*) modulates empathic traits, particularly perspective taking ability.

HTR2A is one of the most abundantly expressed serotonin receptors in the brain, with high levels in the cerebral cortical areas, hippocampus, nucleus accumbens, and caudate nucleus (Barnes and Sharp, 1999). This receptor belongs to the G protein-coupled receptor family and is the primary excitatory receptor of serotonin, mainly acting at post-synaptic neurons. In humans, *HTR2A* is located at 13q14.2. The expression of *HTR2A* is regulated by several functional polymorphisms (Polesskaya and Sokolov, 2002; Myers et al., 2007), among which T102C (rs6313) is the most studied single nucleotide polymorphism in the gene. Compared with the T allele, the C allele leads to lower receptor expressions (Polesskaya and Sokolov, 2002) and lower receptor binding potentials (Turecki et al., 1999), and therefore reduces excitation at post-synaptic neurons (Aghajanian and Marek, 1997).

Although presently there is no study directly investigating the association between *HTR2A* T102C polymorphism and individual differences in empathic traits, a previous study did show that the serotonin neurotransmission, which could be regulated by this polymorphism, plays a role in empathic traits (Nantel-Vivier et al., 2011). This study, focusing on neurotransmitters involved in social interaction and social inference, has indicated that serotonin is crucial for perspective taking, with enhanced serotonin levels relating to better inference of others’ thoughts and feelings in males with a history of aggression. Thus, given the link between *HTR2A* T102C polymorphism and the serotonin levels in the brain and the link between the serotonin levels and perspective taking, it is possible that the *HTR2A* polymorphism modulates an individual’s perspective taking ability. Moreover, previous studies have indicated that the C allele of T102C is associated with poor social functioning in general (Unschuld et al., 2007; Chen et al., 2009; Fraley et al., 2013). The C allele is associated with higher attachment-related anxiety (Fraley et al., 2013), decreased prosocial and affiliative orientations (Unschuld et al., 2007), and less effective aripiprazole treatment on negative symptoms of schizophrenia, including social withdrawal, poor rapport, lack of spontaneity and decreased flow of conversation (Chen et al., 2009). We therefore predict that compared with the T allele, the C allele of T102C is associated with a worse perspective taking ability.

The C allele could also be a risk allele for autism (Smith et al., 2014). A previous family-based study demonstrated that, compared with healthy siblings, autistic individuals have higher incidences of the G allele of A-1438G (i.e., rs6311) in *HTR2A* (Smith et al., 2014). The G allele of A-1438G has an almost complete linkage disequilibrium with the C allele of T102C (Spurlock et al., 1998), with *r^2^* of 0.98 in Caucasian populations (Unschuld et al., 2007) and 1.00 in Asian populations (Chen et al., 2009). We thus predict that individuals with the C allele of T102C are more likely to evidence autistic-like traits. Moreover, the severity of autistic symptoms can be alleviated by perspective taking training (Feng et al., 2008) and impairments in theory of mind/perspective taking ability are underlying causes of social interaction deficits in individuals with autism (Baron-Cohen et al., 1985). Therefore, it is possible that the association between the C allele and autistic-like traits is mediated by one’s ability to take the perspective of others.

## Materials and Methods

### Participants

Five hundred and twenty-three unselected students (388 females, mean age = 24.3 ± 1.4 years) were recruited from Henan University of Science and Technology, China. They were ethnic Han Chinese without any known ancestors of other ethnic origin. All of them were in the normal range of anxiety symptoms (i.e., scores < 50) as assessed by the Zung Self-Rating Anxiety Scale (Zung, 1971; Wang et al., 1999) and of depressive symptoms as assessed by the Zung Self-Rating Depression Scale (Zung, 1965; Wang et al., 1999), except for four participants who had higher scores (51, 51, 57, and 62, respectively) beyond the normal range of depressive symptoms. Given that excluding the four participants did not change the pattern of results, we included them in the following reported data analysis. Written informed consents were obtained from each participant. The study was approved by the Ethics Committee of the Department of Psychology, Peking University and performed in accordance with the Declaration of Helsinki.

### Genotyping

We collected 3–5 hairs with hair follicle cells from each participant and extracted genomic DNA from hair follicle cells by Chelex-100 method (De Lamballerie et al., 1994). This way of DNA sampling is more likely to obtain the consent from the human participants as compared to blood sampling, and less likely to be contaminated as compared to saliva sampling. A 242 bp DNA fragment containing T102C (rs6313) in *HTR2A* gene was produced by polymerase chain reaction (PCR) with the upstream primer, 5′-AACTACGAACTCCCTAA-3′, and the downstream primer, 5′-GTATGTTTCCAGCAAT-3′. The PCR reaction was performed with an initial 5 min denaturation at 94°C, followed by 35 cycles of 94°C for 30 s, 60°C for 30 s, 72°C for 1 min, and a final extension period at 72°C for 5 min. The PCR product was incubated with the restriction enzyme MspI (Fermentas) at 37°C overnight in a 5 μL digestion system, containing 1.0 μL PCR products, 0.40 μL (10U/μL) MspI, 0.40 μL Tango buffer and 3.2 μL H_2_O. The incubated mixture was analyzed by using 8% polyacrylamide gel electrophoresis in 220 V for 2.5 h. The gels were stained with 1.0% silver nitrate solution, and the genotypes bands in gels were identified by the Bio-imaging Systems software. In the current sample of 523 individuals, the distribution of genotypes (CC = 105, CT = 271, TT = 147) did not deviate from Hardy-Weinberg Equilibrium (χ^2^ = 0.970, *p* = 0.325). The genotype frequencies were similar to those found in other Chinese Han samples (Zhang et al., 2008; Chen et al., 2009).

### Interpersonal Reactivity Index (IRI)

Empathic traits were measured with the Chinese version (Rong et al., 2010) of the 28-item IRI (Davis, 1983), which is the most commonly used self-report instrument assessing empathy and which is based on a multidimensional approach. The scale consists of four 7-item subscales including Perspective Taking, Fantasy, Empathic Concern, and Personal Distress. Perspective Taking subscale evaluates an individual’ s cognitive propensity to spontaneously adopt the point of view of others; Fantasy subscale assesses the extent to which people immerse themselves into the feelings and actions of characters in fictional situations; Empathic Concern subscale measures the feeling of warmth, compassion, and concern in response to the misfortune of others; Personal Distress subscale taps “self-oriented” feelings of personal anxiety and discomfort when observing the pain endured by others. For each item, the respondent selected on a 5-point Likert scale the degree to which the description applied to him/herself, with 0 indicating “does not describe me well” and 4 indicating “describes me very well.” The internal consistencies for Perspective Taking, Fantasy, Empathic Concern, and Personal Distress were 0.601, 0.605, 0.642, and 0.684, respectively. They were comparable to what were reported in a previous study (0.59 ≤ α ≤ 0.78) (Rong et al., 2010). The total score of each subscale was calculated according to the scoring procedure suggested by Davis (1983). Of note, 178 participants (out of 523) completed the IRI in a previous study (Gong et al., 2014) and they completed the IRI again for this study.

### Autism-Spectrum Quotient (AQ)

Autistic-like traits were measured with the Chinese version (Liu, 2008) of the 50-item AQ (Baron-Cohen et al., 2001), a self-administered questionnaire for adults of normal intelligence that identifies to what extent the respondent might have features of the core autistic phenotypes. It consists of five 10-item subscales: Social Skill, Attention Switching, Attention to Detail, Communication, and Imagination. Social Skill subscale measures the unwillingness and inability to develop social relationships; Attention Switching subscale assesses the preference to focus on and the inability to shift attention away from stereotyped, repetitive patterns of activities; Attention to Detail subscale evaluates the willingness and ability to notice or remember detail, such as dates and numbers; Communication subscale assesses the unwillingness and inability to initiate or sustain a conversation with others; Imagination subscale measures the unwillingness and inability to engage in symbolic, imaginative activities, including reading fiction, watching dramas, and playing games involving pretending. For each item, the respondent answered ‘definitely agree,’ ‘slightly agree,’ ‘slightly disagree,’ or ‘definitely disagree’ according to the extent to which the description applied to him/herself. Each item scores one point if the respondent agreed with the description of autistic-like behavior, i.e., poor social skill, poor communication, poor imagination, exceptional attention to detail, poor attention switching/strong focus of attention. The internal consistency for the Chinese version of AQ was 0.578 in the present sample, which is slightly lower than the score (0.670) in a previous study with a large healthy sample (Hurst et al., 2007). The total score of each subscale was calculated according to the scoring procedure suggested by Baron-Cohen et al. (2001). For the total score on AQ, eight participants scored from 32 to 34, at or above the cutoff for distinguishing autistic populations versus non-autistic populations (Baron-Cohen et al., 2001). This is to be expected, as autistic traits are reported to a greater extent in the general populations of Eastern cultures than Western cultures (Liu, 2008; Freeth et al., 2013), which strongly suggests that these individuals would not be diagnosed with autism. In addition, the percentage of autistic-like individuals in the current study was consistent with what has been reported in a previous study (Liu, 2008), so we decided to include these participants in the data analysis.

### Statistical Analysis

To test the effects of the *HTR2A* T102C polymorphism on empathic and autistic-like traits, we conducted univariate linear regression analyses with the genotypes (0 = CC, 1 = CT, 2 = TT) as a single predictor (for the outcome variables, see **Table 1**). Because of multiple testing (*n* tests = 10), to control for the rate of false-positive findings by chance, we adjusted *p* values using Bonferroni correction. To estimate the probability of correctly rejecting the null hypothesis when it is false (1 – *β*), *post hoc* power analyses were carried out using the program G^∗^power 3.0 (Faul et al., 2007) with the two-tailed alpha level set at 0.05 (uncorrected) or 0.005 (Bonferroni corrected). The power analysis was also used to calculate the minimal detectable effect. Results indicated that the minimum regression coefficient of 0.122 (i.e., the coefficient of determination *R^2^* > 1.48%) was required for a sample of 523 (two-tailed *α* = 0.05, 1 – *β* = 0.8).

**Table 1:** Results of descriptive statistics and regression analysis examining the effects of *HTR2A* T102C on empathic and autistic-like traits.

Outcome variable	Mean ± (*SD*)	Range	*R*^2^	β	*t*	Uncorrected *p*	Power	Bonferroni corrected *p*	Power after Bonferroni correction	The probability of reaching significance when the subsample size is			
										200 (%)	300 (%)	400 (%)	500 (%)
**IRI**													
Perspective Taking	17.7 ± 3.9	5–28	0.017	0.130	3.002	0.003	0.849	0.028	0.571	44	67	91	100
Fantasy	17.9 ± 4.2	6–28	<0.001	-0.006	-0.147	0.883	0.052	1.000	0.005	1	0	0	0
Empathic Concern	21.2 ± 3.6	4–28	<0.001	0.016	0.354	0.723	0.065	1.000	0.008	2	1	0	0
Personal Distress	14.6 ± 4.6	0–26	0.015	-0.123	-2.829	0.005	0.808	0.049	0.507	39	60	84	100
**AQ**													
Social Skill	4.2 ± 2.4	0–10	0.004	-0.064	-1.458	0.146	0.310	1.000	0.089	9	9	8	1
Attention Switching	5.0 ± 1.8	0–10	0.007	-0.081	-1.860	0.063	0.458	0.635	0.170	15	20	25	24
Attention to Detail	4.4 ± 2.1	0–10	0.013	0.114	2.616	0.009	0.745	0.092	0.424	34	51	73	100
Communication	3.1 ± 1.9	0–9	0.016	-0.125	-2.880	0.004	0.820	0.041	0.525	41	62	86	100
Imagination	4.0 ± 1.6	0–9	<0.001	0.002	0.046	0.963	0.050	1.000	0.005	1	0	0	0
Total Score	20.8 ± 5.0	8–34	0.003	-0.059	-1.343	0.180	0.271	1.000	0.072	7	7	5	0

*We conducted regression analysis with the genotypes (0 = CC, 1 = CT, 2 = TT) as a single predictor of each outcome variable. β refers to the regression coefficient of the genotypes; *t* refers to the *t*-statistic of β; uncorrected *p* refers to the significance of β before Bonferroni correction; Bonferroni corrected *p* refers to the significance of β after Bonferroni correction. The probability of reaching significance refers to the probability of the estimated regression coefficients reaching significance in the 20,000 subsamples. The size of the subsample was set at 200, 300, 400, or 500.*

To test the robustness of the results in the regression analyses, we randomly selected a subsample with a given size (e.g., *N* = 400) from the total sample 20,000 times and estimated the regression coefficient in each simulated subsample. Then we calculated the probability of the estimated regression coefficients reaching significance in the 20,000 subsamples. The size of the subsample was set at 200, 300, 400, or 500 (**Table 1**).

To test for the mediating role of perspective taking in the association between the *HTR2A* T102C polymorphism and communication problems, we bootstrapped the indirect effect of the polymorphism on Communication through Perspective Taking 20,000 times using the SPSS version of INDIRECT macro (http://www.afhayes.com/; Preacher and Hayes, 2008) and obtained the bias-corrected 95% confidence interval of the indirect effect. The indirect effect is considered statistically significant at *p* < 0.05 when the 95% confidence interval does not include zero.

In our previous study, we found an association between a variant (-1021C/T) in the *DBH* gene and empathic traits, with the CC carriers showing higher scores on the Empathic Concern subscale than the CT/TT carriers (Gong et al., 2014). To examine whether the effects of the T102C polymorphism on empathic and autistic-like traits continued to hold after controlling for the scores on Empathic Concern subscale and/or the -1021C/T polymorphism in the *DBH* gene, we conducted hierarchical regression analysis: step 1, entering control variables; step 2, entering both control variables and the T102C polymorphism.

## Results

### Direct Effect

**FIGURE 1:**
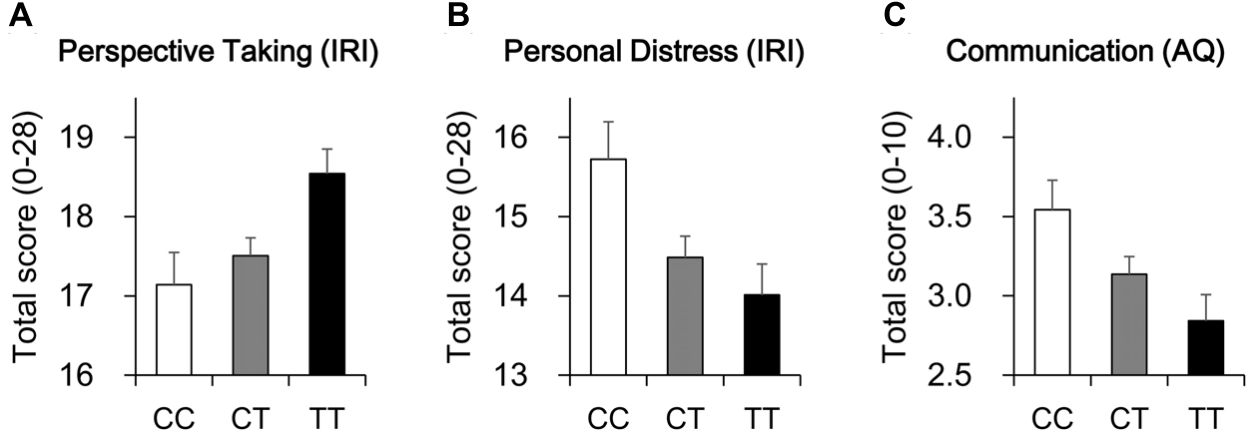
**Effects of *HTR2A* T102C polymorphism on perspective taking and autistic-like traits. (A)** Individuals with a greater number of the C alleles, which is associated with lower activity of HTR2A, were less likely to spontaneously adopt the point of view of others. **(B)** Individuals with a greater number of the C alleles were more likely to be anxious when observing the pain endured by others. **(C)** Individuals with a greater number of the C alleles were more likely to have communication problems.

As shown in **Table 1**, regression analyses revealed that the polymorphism was significantly associated with the total scores on the Perspective Taking subscale and Personal Distress subscale of IRI, and the Communication subscale of AQ both before and after Bonferroni correction. Individuals with a greater number of the C alleles, which is associated with lower activity of HTR2A, were less likely to spontaneously adopt the point of view of others, were more likely to be anxious when observing the pain endured by others, and were more likely to have communication problems (**Figure 1**). Additionally, results also showed that the polymorphism seemed to be associated with scores on the Attention to Detail subscale of AQ, with a greater number of the C alleles significantly associating with a decreased ability to notice or remember details; however, this result did not survive Bonferroni correction (**Table 1**).

### Mediation Analysis

**FIGURE 2:**
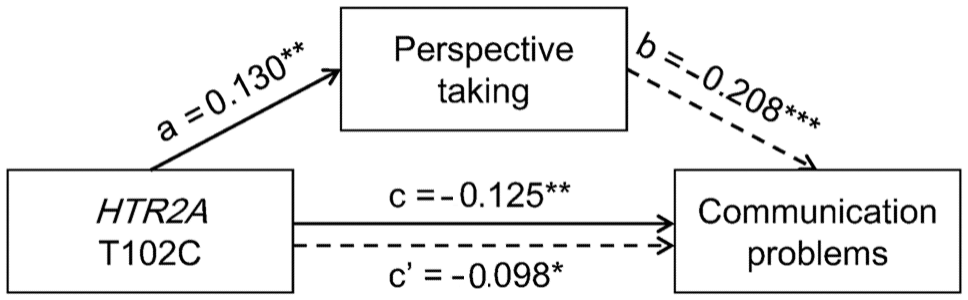
**The mediating role of perspective taking ability in the association between *HTR2A* T102C polymorphism (0 = CC, 1 = CT, 2 = TT) and communication problems.** All estimates are standardized, ^∗^*p* < 0.05, ^∗∗^ *p* < 0.01, ^∗∗∗^ *p* < 0.001.

Considering that previous studies have demonstrated the causal link between individuals’ perspective taking ability and communication problems (Saxton et al., 2013; Wardlow et al., 2014), we conducted a mediation analysis to examine whether the genotype effect on communication would be mediated by individuals’ perspective taking abilities. Compared with a regression in which genotype was included as the only predictor of Communication (the genotype effect: *β* = -0.125, *t* = -2.880, *p* = 0.004), when both genotype and Perspective Taking were included as predictors, the effect of genotype on Communication decreased, *β* = -0.098, *t* = -2.285, *p* = 0.023 (**Figure 2**). The total indirect effect accounted for 21.6% (1-0.098/0.125) of the genotype effect on Communication. Mediation analysis indicated a significant mediating effect of perspective taking ability on the relationship between *HTR2A* T102C polymorphism and communication problems, indirect effect estimate = -0.0075, *SE* = 0.0031, bias-corrected 95% confidence interval is [-0.0150, -0.0026].

### Supplementary Analysis

Given the association between a variant (-1021C/T) in the *DBH* gene and empathic traits observed in our previous study (Gong et al., 2014), we included this variant and scores on Empathic Concern in hierarchical regression analysis and found that the effects shown in **Figures 1** and **2** remained significant after controlling for scores on Empathic Concern and/or the variant in the *DBH* gene (**Tables 2** and **3**).

**Table 2:** Results of hierarchical regression analysis examining the effects of the T102C polymorphism on empathic and autistic-like traits after controlling for the scores on Empathic Concern subscale and/or the -1021C/T polymorphism (0 = CT/TT, 1 = CC) in the *DBH* gene.

Outcome variable	Control variable(s)	*R^2^* change	*F* change	*β*	Uncorrected *p*
IRI Perspective Taking	IRI Empathic Concern	0.016	8.992	0.127	0.003
	-1021C/T polymorphism	0.020	10.368	0.142	0.001
	IRI Empathic Concern and the -1021C/T polymorphism	0.018	9.999	0.136	0.002
IRI Personal Distress	IRI Empathic Concern	0.016	8.618	-0.126	0.003
	-1021C/T polymorphism	0.016	8.149	-0.126	0.004
	IRI Empathic Concern and the -1021C/T polymorphism	0.017	9.025	-0.130	0.003
AQ Communication	IRI Empathic Concern	0.015	8.175	-0.124	0.004
	-1021C/T polymorphism	0.016	8.375	-0.127	0.004
	IRI Empathic Concern and the -1021C/T polymorphism	0.016	8.145	-0.125	0.004

*R^2^ change refers to the change of the determinism coefficient between the models in step 1 and in step 2. *F* change refers to the change of the *F* value between the models in step 1 and in step 2. β refers to the regression coefficient of the T102C polymorphism; uncorrected *p* refers to the significance of β without correction.*

**Table 3:** Results of mediation analysis after controlling for the scores on Empathic Concern subscale and/or the -1021C/T polymorphism in the *DBH* gene.

Control variable(s)	Indirect effect estimate	*SE*	Bias-corrected 95% confidence interval
IRI Empathic Concern	-0.0071	0.0030	[-0.0145, -0.0024]
-1021C/T polymorphism	-0.0083	0.0032	[-0.0161, -0.0032]
IRI Empathic Concern and the -1021C/T polymorphism	-0.0077	0.0031	[-0.0154, -0.0029]

Of note, 178 out of 523 participants completed the IRI previously in another study (Gong et al., 2014) and they completed the IRI again in the present study. We also examined the effects of the T102C polymorphism on empathic traits after excluding the data of the 178 participants or replacing these data with the scores in their first test. The pattern of the effects on the four subscales of IRI continued to hold (**Table 4**).

**Table 4:** Results of regression analysis examining the effects of *HTR2A* T102C on empathic traits after excluding the 178 participants who participated Gong et al. (2014) or replacing these data with the scores in their first test.

Outcome variable	Mean ± (*SD*)	Range	*R*^2^	β	*t*	Uncorrected *p*
**Excluding the 178 participants**					
Perspective taking	17.8 ± 3.9	5–28	0.027	0.164	3.072	0.002
Fantasy	17.8 ± 4.4	7–28	<0.001	-0.003	-0.061	0.951
Empathic concern	21.0 ± 3.7	4–28	0.001	0.031	0.568	0.571
Personal distress	14.7 ± 4.6	4–26	0.019	-0.138	-2.572	0.011
**Replacing the data with the scores in their first test**					
Perspective taking	17.7 ± 3.9	5–28	0.016	0.127	2.926	0.004
Fantasy	17.8 ± 4.3	6–28	<0.001	-0.022	-0.509	0.611
Empathic concern	21.0 ± 3.6	4–28	0.003	0.054	1.226	0.221
Personal distress	14.8 ± 4.6	3–26	0.015	-0.123	-2.839	0.005

Moreover, results from our simulated subsamples revealed that the effects shown in **Figure 1** had increased probabilities of reaching significance as the subsample size increased (**Table 1**); the probability of reaching significance was 84–91% when the subsample size set at 400.

## Discussion

The current study investigated to what extent *HTR2A* T102C polymorphism modulates individuals’ empathic and autistic-like traits. As predicted, we found that the individuals with a greater number of the C alleles, which is associated with lower activity of HTR2A, were less likely to spontaneously adopt the point of view of others and more likely to be anxious when observing the pain endured by others. We also found that individuals with a greater number of the C alleles were more likely to have communication problems, an autistic-like trait. Further analysis showed that the genotype effect on communication is mediated by individuals’ perspective taking abilities.

Previous studies have demonstrated that the reduced availability of HTR2A impairs empathy-related behaviors, such as social communication (Murphy et al., 2006) and prosocial and affiliated orientations (Gerretsen et al., 2010). The present study extended these findings by suggesting that the serotonin receptor gene, *HTR2A*, which regulates the serotonin levels in the brain, to a certain extent, is associated with individual differences in empathic traits. Individuals with a greater number of the C alleles of *HTR2A* T102C polymorphism, which is related to reduced excitation at post-synaptic neurons (Aghajanian and Marek, 1997), showed decreased perspective taking ability and increased personal distress. On the surface, these findings are similar to those reported in two recent studies showing the genotype effects of the promoter region (5-HTTLPR) of the serotonin transporter gene (*5-HTT*) on self-reported empathic traits (Pełka-Wysiecka et al., 2012) and on physiological responses when observing others in distress (Gyurak et al., 2012). However, HTR2A and 5-HTT may have different mechanisms of regulating the serotonin availability, considering their differential distributions in pre- and post-synaptic membranes. HTR2A receptors are mainly distributed at post-synaptic membranes, whereas 5-HTT proteins are mainly distributed at pre-synaptic membranes (Zhou et al., 1998). The effect of *HTR2A* gene on empathy may result from the role of T102C in spontaneous excitatory postsynaptic potentials while the effect of *5-HTT* gene on empathy may be attributed to the impact of 5-HTTLPR on the reuptake of serotonin into pre-synaptic membranes.

Consistent with the previous study suggesting the role of *HTR2A* T102C polymorphism in the devolvement of autistic symptoms (Smith et al., 2014), we found that the polymorphism underlies individual differences in autistic-like traits in normal adults. This finding extends our knowledge about the impact of this polymorphism on autistic symptoms, particularly communication problems, both in individuals diagnosed with autism to individuals who do not meet the criteria for autism/healthy populations. A previous study indicated that among the subscales of AQ, communication problems and social skills in autistic-like traits differentiated the parents with autistic children from those without autistic children (Bishop et al., 2004). It is possible that the association between the C allele and communication problems is one of the causes underlying the increased incidences of the C allele in autistic children.

In line with the previous studies showing the association between the C allele and poor social functioning (Unschuld et al., 2007; Fraley et al., 2013), this study found that the C allele was associated with autistic-like traits and impaired perspective taking ability. Our findings implicate the overlapping genetic basis underlying the individual differences in empathic traits and autistic symptoms. Moreover, our mediation analysis showed that perspective taking mediated the association between *HTR2A* T102C polymorphism and communication problems. Perspective taking ability may not only promote communication skills through facilitating the accuracy of predicting others’ behaviors and reactions (Tanida and Yamagishi, 2004; Galinsky et al., 2008) but may also increase the willingness to communicate with others by reducing the stereotype and prejudice against others (Galinsky et al., 2008; Wang et al., 2012). It is thus possible that individuals with a greater number of the C allele are more likely to misunderstand the intentions and behaviors of others and more likely to have difficulties in communicating with others. Our findings suggest a possible psychobiological mechanism underlying the genotype effect on deficits in social interactions.

Several limitations of this study should be noted. First, we demonstrated small effects of a single polymorphism on empathic and autistic traits while these traits are likely to be influenced by multiple polymorphisms (Rodrigues et al., 2009; Wu et al., 2012; Gong et al., 2014) and by a variety of non-genetic factors (O’Reilly and Peterson, 2014). More systematic studies are needed to simultaneously take into consideration genetic and environmental factors underscoring individual differences in empathic and autistic traits. Second, the present study relied on self-report questionnaires which require the participants to be introspective to provide accurate response to the items. The lack of introspective ability and the influence of social desirability may add noises to the measurement, thereby leading to over- or under-estimation of the contribution of the *HTR2A* T102C polymorphism to individuals’ empathic and autistic traits. Third, our power analyses suggested that a genotype effect accounting for less than 1.48% of the phenotypic variance would not be detected because of the medium sample size (*N* = 523) in the present study. As the effect size of a single polymorphism for a complex trait is relatively small (Lander, 2011; Hewitt, 2012), this study might suffer from the low statistical power to distinguish some small but true effects from random chance. Finally, the findings do not have a direct replication in a second sample, a recommended procedure for screening false findings in candidate gene association studies (Hewitt, 2012). To substantiate our claims, we randomly selected subsamples from the total sample and calculated the probability of obtaining significant effects in these samples. We found that our findings have a high probability to be replicated in subsamples of enough size. We have also applied Bonferroni correction to control for false positives in multiple testing.

## Conclusion

By differentiating individuals according to the polymorphism of *HTR2A* T102C and by measuring empathic and autistic-like traits with IRI and AQ scale, we demonstrated the impact of *HTR2A* gene on individuals’ empathic traits and social communication in a general population. Our findings highlight the role of the *HTR2A* gene in social functioning and the connection between perspective taking and autistic-like traits.

## Author Contributions

PG and JL contributed equally to this work. PG, JL, and XZ designed the study. PG, JL, PB, and XZ wrote the manuscript. PG, JL, and SL performed the experiment and analyzed the data.

## Conflict of Interest Statement

The authors declare that the research was conducted in the absence of any commercial or financial relationships that could be construed as a potential conflict of interest.
